# KDM5 family as therapeutic targets in breast cancer: Pathogenesis and therapeutic opportunities and challenges

**DOI:** 10.1186/s12943-024-02011-0

**Published:** 2024-05-20

**Authors:** Chang-Yun Li, Wanhe Wang, Chung-Hang Leung, Guan-Jun Yang, Jiong Chen

**Affiliations:** 1 State Key Laboratory for Managing Biotic and Chemical Threats to the Quality and Safety of Agro-products, Ningbo University, Ningbo, 315211 Zhejiang China; 2 Laboratory of Biochemistry and Molecular Biology, School of Marine Sciences, Ningbo University, Ningbo, 315211 China; 3 Institute of Medical Research, Northwestern Polytechnical University, Xi’an, Shaanxi 710072 China; 4 State Key Laboratory of Quality Research in Chinese Medicine, Institute of Chinese Medical Sciences, University of Macau, Macau, China; 5 Department of Biomedical Sciences, Faculty of Health Sciences, University of Macau, Macau, China; 6 Macao Centre for Research and Development in Chinese Medicine, University of Macau, Macau, China; 7 MoE Frontiers Science Centre for Precision Oncology, University of Macau, Macau, China

**Keywords:** Breast cancer, KDM5, Histone demethylation, Therapeutic target, KDM5 inhibitors

## Abstract

Breast cancer (BC) is the most frequent malignant cancer diagnosis and is a primary factor for cancer deaths in women. The clinical subtypes of BC include estrogen receptor (ER) positive, progesterone receptor (PR) positive, human epidermal growth factor receptor 2 (HER2) positive, and triple-negative BC (TNBC). Based on the stages and subtypes of BC, various treatment methods are available with variations in the rates of progression-free disease and overall survival of patients. However, the treatment of BC still faces challenges, particularly in terms of drug resistance and recurrence. The study of epigenetics has provided new ideas for treating BC. Targeting aberrant epigenetic factors with inhibitors represents a promising anticancer strategy. The KDM5 family includes four members, KDM5A, KDM5B, KDM5C, and KDMD, all of which are Jumonji C domain-containing histone H3K4me2/3 demethylases. KDM5 proteins have been extensively studied in BC, where they are involved in suppressing or promoting BC depending on their specific upstream and downstream pathways. Several KDM5 inhibitors have shown potent BC inhibitory activity in vitro and in vivo, but challenges still exist in developing KDM5 inhibitors. In this review, we introduce the subtypes of BC and their current therapeutic options, summarize KDM5 family context-specific functions in the pathobiology of BC, and discuss the outlook and pitfalls of KDM5 inhibitors in this disease.

## Introduction

Breast cancer (BC) is a growing health issue worldwide, with 2.3 million new diagnoses and 685,000 deaths reported in 2020 [1]. Today, BC has become the leading malignant tumor accounting for nearly 12% of all new cancer diagnoses worldwide according to the WHO. In China, the annual incidence has increased by 3-4% per year in recent years. Developing precise strategies for the prevention and therapy of BC can alleviate the suffering of both BC patients and individuals with potential risk.

Cancer is simultaneously a genetic disease and an epigenetic disease. Although epigenetics does not alter the DNA sequence, it influences the pathogenesis of cancer at the gene and protein levels [2]. Epigenetics regulates cell proliferation, differentiation, metabolism, metastasis, and the microenvironment by inducing reversible alterations in the chromatin landscape [3]. The study of epigenetics for cancer diagnosis, therapy, and prognosis has received significant attention in recent years [4]. Targeting epigenetic regulatory factors as an adjuvant strategy for chemotherapy holds great promise in improving treatment precision. A diversity of inhibitors targeting epigenetic modifying enzymes, such as histone deacetylase (HDAC) and DNA methyltransferase, have received FDA approval, while numerous drugs are also undergoing clinical trials [5, 6].

The occurrence and progression of BC as well as the development of drug resistance are closely linked to epigenetic abnormalities [7]. For example, dysregulation of DNA methylation can promote or maintain the cancer cell stemness, thereby contributing to the pathogenesis of BC [8, 9]. Moreover, TNBC patients exhibited widespread genomic hypomethylation, while the buildup of methylation enhanced the risk of BC in postmenopausal women [10, 11]. In addition, dysregulation of histone modification also serves as a crucial marker in cancer [12, 13]. HDACs are involved in breast cancer progression by regulating the stemness, and invasion, metastasis of cancer cells [14]. Clinical evidence indicates that histone 3 lysine 4 di- and tri-methylation (H3K4me2/3) levels are raised in cancers of the breast and colon, which is associated with an unfavorable prognosis [13, 15].

**Fig. 1:**
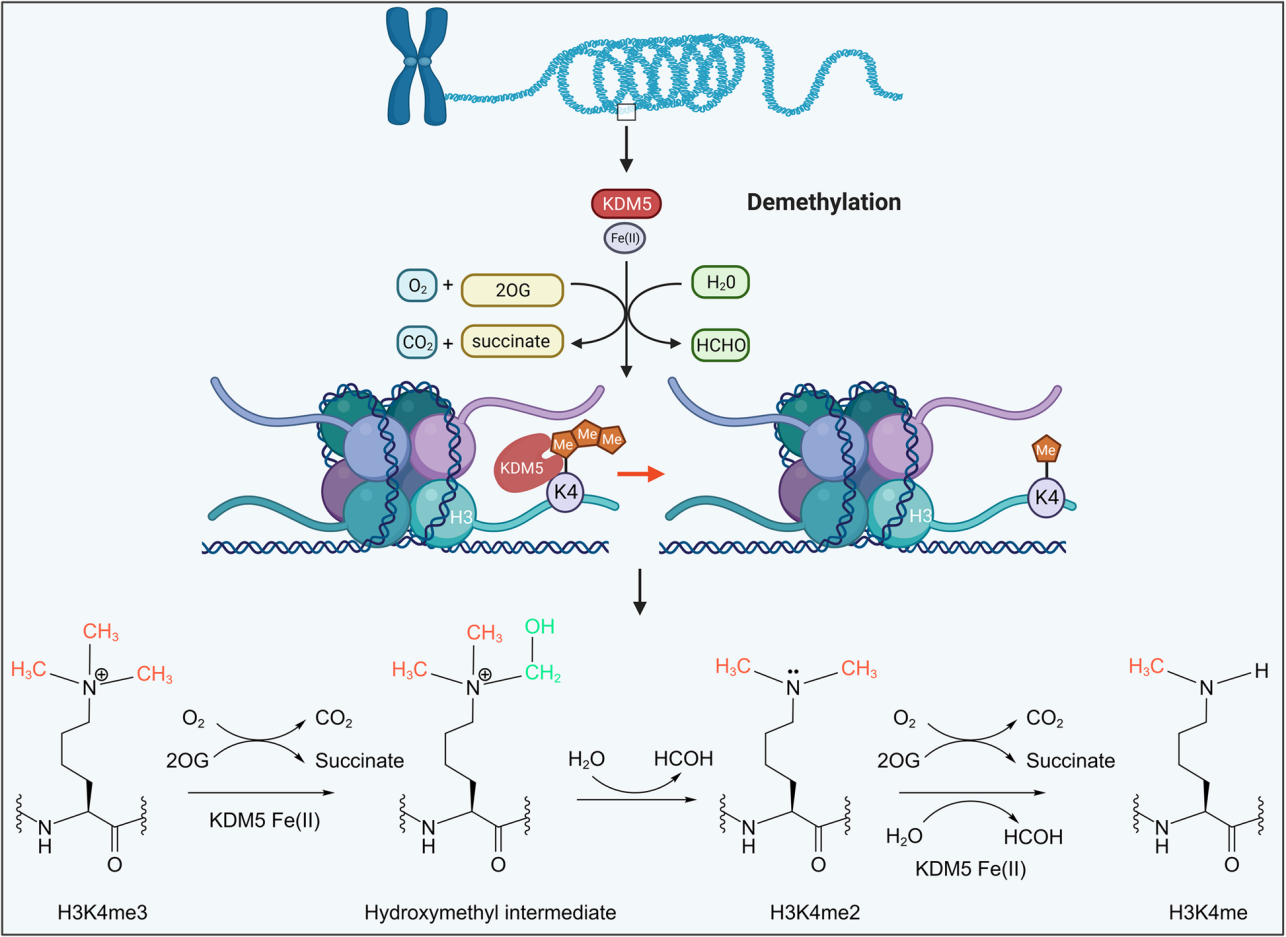
Histone demethylation mechanism of KDM5. KDM5 proteins recognize and bind histone tails H3K4me2/3 in chromosomes and then catalyze hydroxylation of methyl groups in the JmjC domain with the assistance of cofactors Fe(II) and 2-OG, followed by hydroxymethyl cleavage to form formaldehyde and produce lysine with one fewer methyl group

Histone lysine demethylation is a common histone modification with important functions on chromatin structure catalyzed by histone lysine demethylases (KDMs). KDMs consist of two classes of proteins of over 30 members, including the (i) flavin-dependent monoamine oxidases (LSD), and (ii) the Fe(II)- and α-ketoglutarate (2OG)-dependent oxygenases that possess a conserved catalytic Jumonji C domain (JmjC) [16]. Many KDMs members, such as LSD1, KDM4A/B/C/D, KDM6A, and KDM7A, are associated with BC progress in a subtype/content-dependent manner, and inhibitors targeting them have been extensively developed [16–21]. KDM5, also known as JARID1 (jumonji domain ARID-containing protein), belongs to the JmjC family and comprises the four members KDM5A/B/C/D, which are encoded in the human genome at loci 12p13.33, 1q32.1, Xp11.22, and Yq11.223.1, respectively. KDM5 proteins can remove H3K4me2/3 marks, which function as markers for transcriptional activation. The catalytic mechanism of demethylation is similar to that exhibited by other members of the JmjC family, primarily involving the oxidation of Fe(II), decarboxylation of 2-OG, and formation and cleavage of lysine hydroxymethyl groups (Fig. 1) [22].

**Fig. 2:**
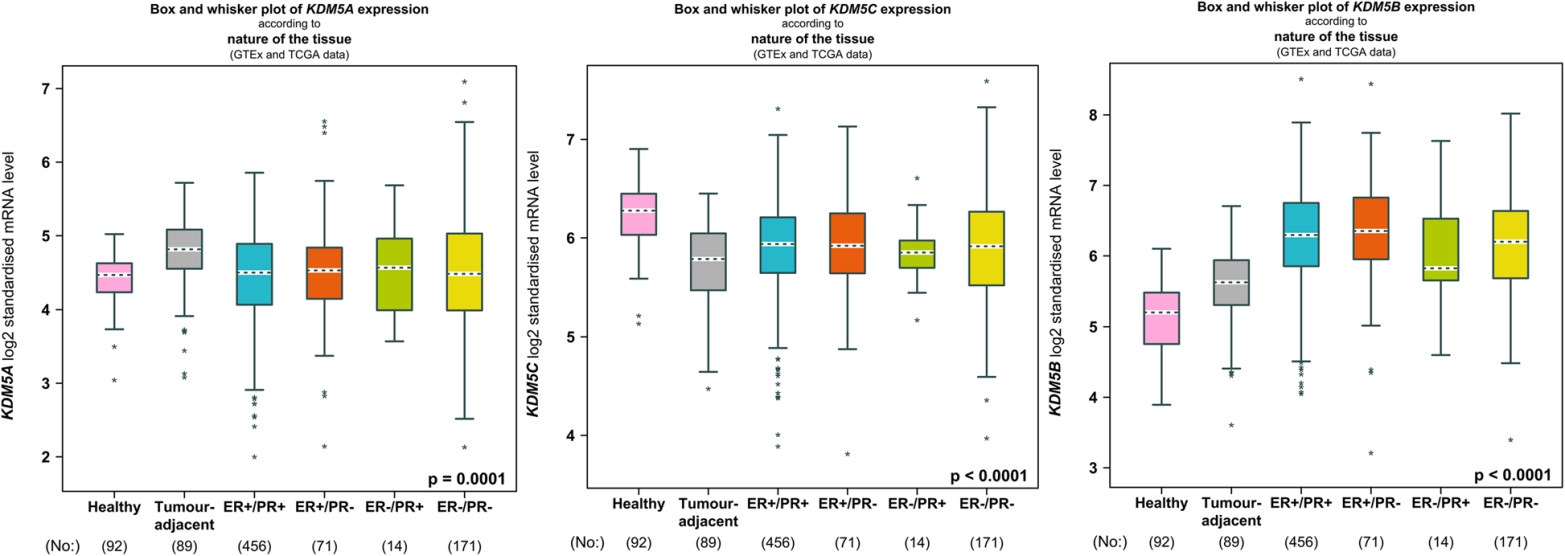
Expression analysis of KDM5 family in BC. *KDM5A/B* exhibit higher expression levels in breast cancer tissues compared to normal tissues, while *KDM5D* is not expressed in the female mammary gland

In BC, KDM5 family members especially KDM5A/B are often overexpressed and promote tumor development by regulating cellular and molecular mechanisms (Fig. 2). Therefore, a deeper understanding of how KDM5s regulate BC progression is essential for the discovery of new therapies to reduce drug resistance and metastasis. In this review, we provide a summary of the regulatory systems governing KDM5 proteins during BC development, metastasis, and drug resistance, and discuss the current status of KDM5 inhibitors in BC treatment, to provide insights for the innovation of BC therapeutic strategies targeting KDM5 family members.

## Breast cancer and drugs

### Breast cancer

**Fig. 3:**
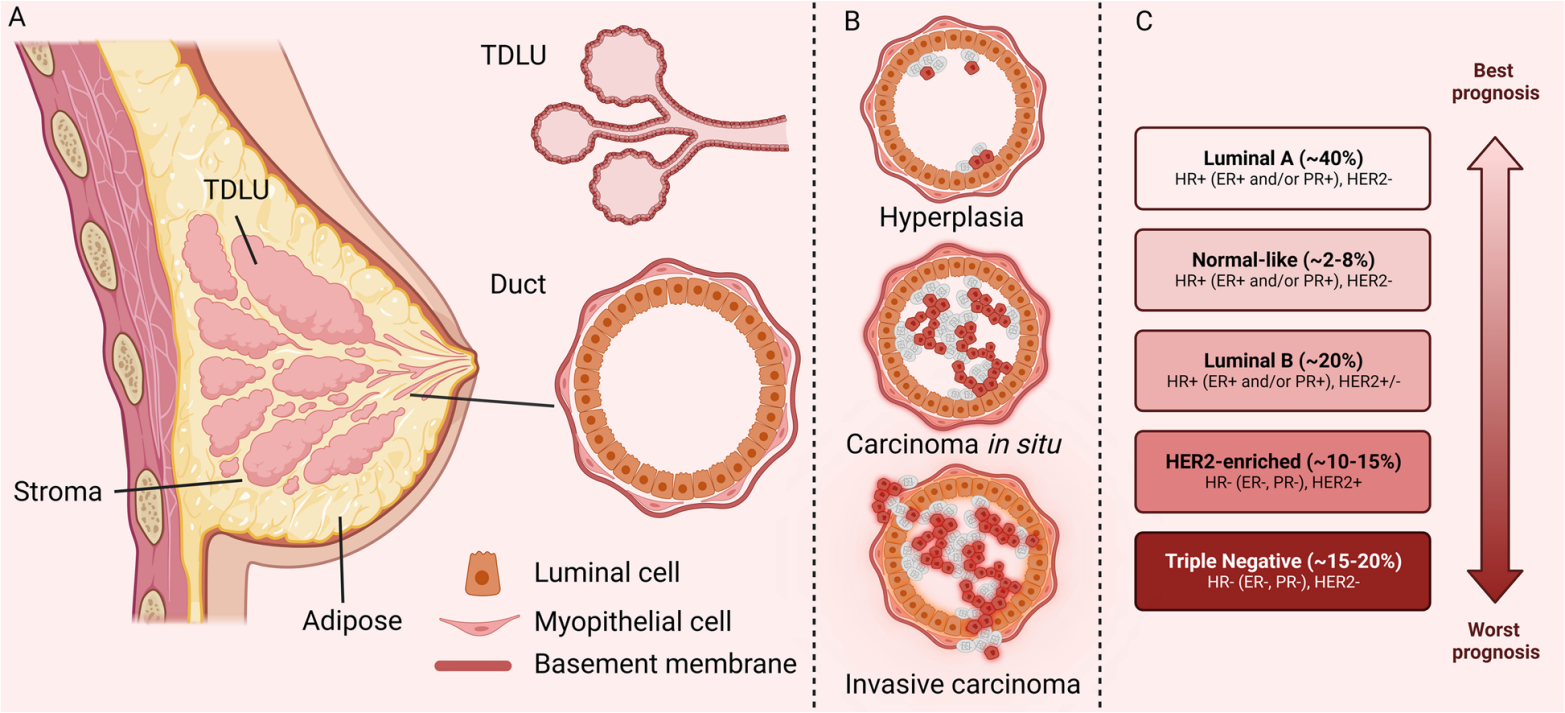
The structure of the mammary gland and the origin of BC cells. (**A**) The breast has 15–25 mammary lobes, each of which is an independent gland. Terminal ductal lobular unit (TDLU) is the primary anatomical source of most BC precursors and cancers. Mammary ducts are composed of luminal and myoepithelial cells surrounded by basement membranes. (**B**) About 5% of breast hyperplasia will develop into BC in situ. If the cancer cells break through the basement membrane of the ducts or lobular, they will spread to the surrounding tissues and form invasive BC. (**C**) Clinical subtypes of BC and their percentage

Approximately 80% of BCs originate from the epithelial cells of the duct, while an additional 15% arise from the lobule in the mammary gland [23]. From a histological perspective, BCs can be classified as carcinoma in situ (CIS) or invasive carcinoma. CIS is restricted to the ducts and lobules and has not yet attacked the breast tissue, but its progression can lead to invasive carcinoma [24]. The main subtypes of invasive BC are invasive ductal, invasive lobular, and mixed ductal/lobular as well as others [25]. Clinically, BC is broadly categorized into three subtypes depending on the status of hormone receptors and growth factors: estrogen and progesterone receptor positive (ER **+** and PR**+**, 70%), human epidermal growth factor receptor 2 positive (HER2+, 15–20%), and triple-negative BC (TNBC, 10–15%) (Fig. 3) [26, 27]. Another subtyping system based on gene expression analysis includes five categories, namely normal-like, luminal A, luminal B, HER2, and basal-like [28]. The luminal subtype is characterized by hormone receptor positivity, while the Ki-67 proliferation marker can be used to differentiate between luminal A (low) and B (high). Luminal A is more frequent than luminal B and has a lower histological grade as well as a superior prognosis [29], while HER2 is more aggressive than luminal B [30]. The basal-like subtype has a high Ki67 index and exhibits phenotypic similarities with TNBC [31]. As TNBC is more aggressive and lacks a signature receptor, TNBC has a greater recurrence probability and a worse 5-year survival rate compared to other types of BC.

More than 90% of BC patients do not exhibit metastasis at diagnosis, emphasizing the key treatment objective as complete tumor eradication and prevention of future occurrences [32]. The most frequent regions of metastasis for BC patients are the bones, lungs, brain, and liver [33]. As metastatic BC is a primary factor of death among BC patients, the objective of treatment is to lengthen survival duration and improve the quality of life of patients.

BC cell lines have been widely used in both in vitro and in vivo biological research. Understanding the molecular characteristics of BC cell lines is crucial for the accurate selection of appropriate cell lines in preclinical investigations targeting distinct subtypes of BC. Commonly used BC cell lines are summarized in Table 1 [34–36].

**Table 1:** Molecular information and categorization of breast cancer cell lines

Cell lines	ER	PR	HER2	BRCA1 Mutation	Subtype
MCF-7	+	+	-	Wild type	Lumina A
ZR-75-1	+	+/-	-	Wild type	Lumina A
T-47D	+	+	-	Wild type	Lumina A
EFM-19	+	+	-	Wild type	Lumina A
EVSA-T	-	+	-	Wild type	Lumina A
ZR-75-30	+	-	+	Wild type	Lumina B
BT-474	+	+	+	Wild type	Lumina B
UACC-812	+	+/-	+	Wild type	Lumina B
SK-BR-3	-	-	+	Wild type	HER2
HH315	-	-	+	Wild type	HER2
HCC1954	-	-	+	Wild type	HER2
MDA-MB-231	-	-	-	Wild type	TNBC
HCC1937	-	-	-	Mutation	TNBC
SUM149	-	-	-	Mutation	TNBC
SUM102	-	-	-	Wild type	TNBC
BT-549	-	-	-	Wild type	TNBC
MDA-MB-468	-	-	-	Wild type	TNBC
MDA-MB-453	-	-	-	Wild type	TNBC
HCC70	-	-	-	Wild type	TNBC

*+: *Positive, -:Negative

The majority of women encounter side effects during conventional BC treatment, which not only hinders treatment completion but also compromises its potential benefits [37]. With a deeper understanding of BC, more drugs and therapies are constantly being developed to alleviate the suffering of patients. BC treatment strategies can encompass a comprehensive approach involving chemotherapy, endocrine therapy, targeted therapy, immunotherapy, surgical intervention, and radiation therapy [32]. The subtypes and stages of BC as well as the individualized needs of patients should be considered when formulating treatment strategies.

### Drugs for breast Cancer

**Fig. 4:**
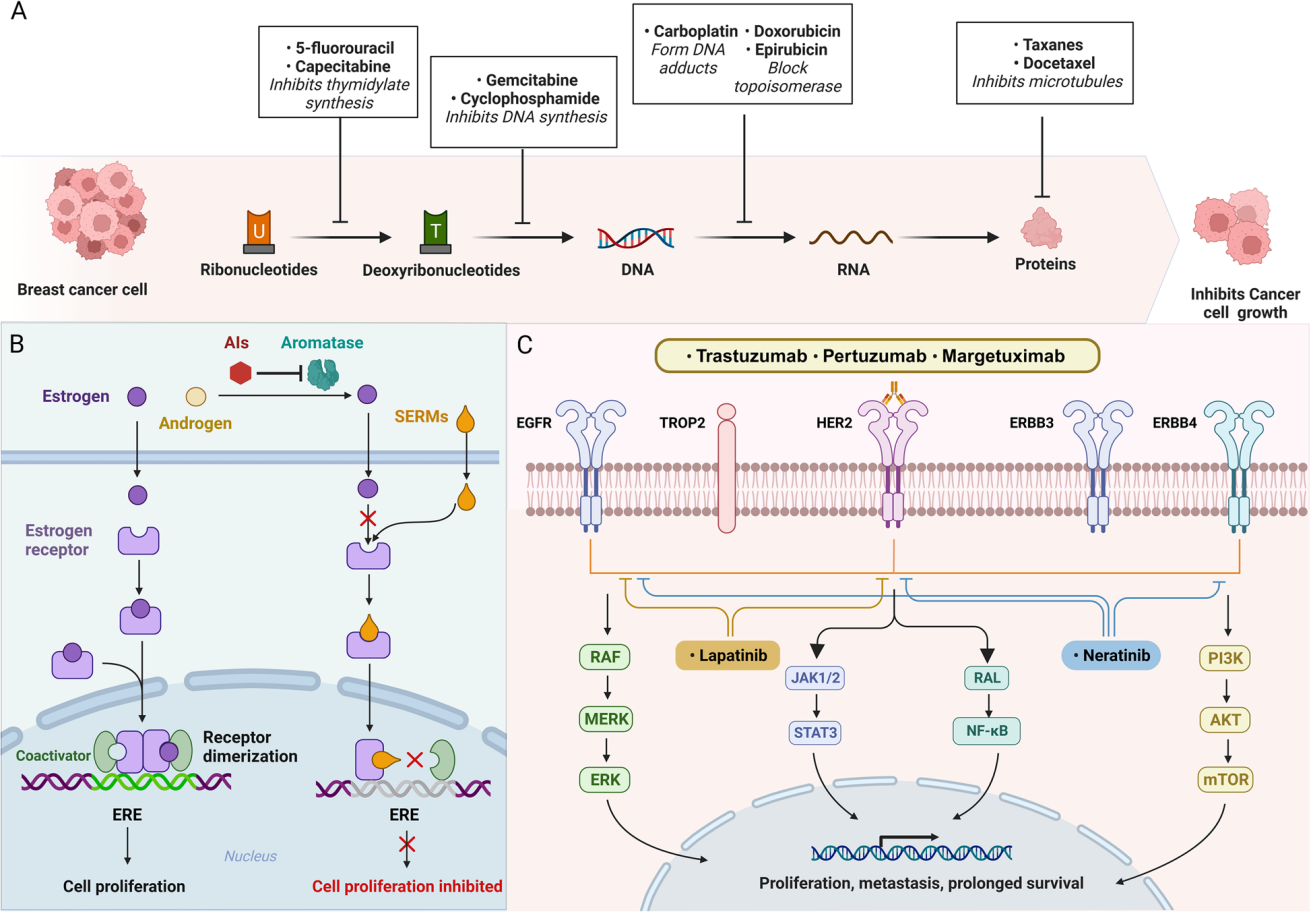
Currently used drugs for treating BC and their therapeutic mechanisms. (**A**) Chemotherapeutic agents are a commonly used treatment for cancer that inhibit cancer cell growth and exert cytotoxicity by acting at multiple levels of DNA, RNA, proteins, and metabolites. (**B**) Selective estrogen receptor modulators (SERMs) and aromatase inhibitors (AIs) hinder estrogen receptor dimerization and thus inhibit target genes activation by competing for binding to the estrogen receptor and inhibiting estrogen production, respectively. (**C**) Monoclonal antibodies targeting HER2 extracellular surface structures are commonly used in HER2-positive BC to inhibit HER2 activation. Some small molecule tyrosine kinase inhibitors inhibit the HER2 signaling cascade amplification within cells

Currently, multiple FDA-approved drugs for BC are administered as neoadjuvant or adjuvant therapies [38]. Chemotherapy impedes the proliferation of cancers by damaging DNA, preventing DNA synthesis, and disrupting cell division [39]. At the same time, chemotherapy can also affect non-cancer cells, and common adverse reactions include myelosuppression, nausea, and vomiting [40]. Anthracyclines doxorubicin and epirubicin have been widely used in the treatment of BC due to their cytotoxic and anti-proliferative effects. The mechanism of these drugs includes inducing apoptosis by inhibiting the topoisomerase II and causing DNA damage through intercalation between DNA double helix bases (Fig. 4) [28]. Paclitaxel and doxorubicin are anti-microtubule drugs commonly used in BC chemotherapy, which impede cell division by promoting microtubule polymerization, stabilizing polymerized microtubules, and interfering with microtubule depolymerization [41]. Cyclophosphamide is one of the commonly used drugs in adjuvant chemotherapy for BC. It is a pro-drug that is catalyzed by CYP450 into metabolites that have alkylating properties, resulting in inhibition of DNA synthesis and induction of apoptosis [42]. Antimetabolites are structurally similar to normal metabolites in the body and play an antitumor role by interfering with nucleic acid synthesis via inhibiting enzymes necessary for metabolites [43]. Common antimetabolites for BC include 5-fluorouracil (a fluorinated derivative of uracil), capecitabine (an oral precursor of 5-fluorouracil), and gemcitabine (a pyrimidine nucleotide analog) [44, 45]. Platinum-based drugs (e.g. carboplatin) that can damage the DNA of tumor cells are more effective in TNBC patients especially those with *BRCA* mutations [46].

The main treatment for hormone receptor-positive BC is endocrine therapy, which includes selective estrogen receptor modulators, and aromatase inhibitors (Fig. 4) [32]. Estrogen receptor modulators compete with estradiol to bind to estrogen receptors, inhibiting estrogen signaling and BC cell proliferation. Commonly used drugs in this category include tamoxifen, toremifene, and raloxifene [47]. Aromatase inhibitors block the conversion of androgens to estrogens by inhibiting aromatase activity, thereby decreasing the levels of estrogen. In the treatment of estrogen-dependent BC, drugs such as exemestane, letrozole, and anastrozole have shown promising therapeutic effects [48].

The introduction of targeted HER2 therapy has revolutionized the landscape of BC treatment. HER2 is a transmembrane tyrosine kinase receptor that regulates the proliferation, differentiation, angiogenesis, invasion, and metastasis of cancer cells [49]. Trastuzumab is a humanized monoclonal antibody that targets the extracellular epitope of HER2 (Fig. 4) [50]. By binding to HER2, trastuzumab induces downregulation of receptor signals thus inhibiting the progression of HER2-overexpressed BC [51]. Pertuzumab is also a monoclonal antibody that targets HER2 domain II and blocks heterodimerization of HER2 and HER3 [52]. Margetuximab is a Fc genetically engineered monoclonal antibody that binds to the same epitope as trastuzumab but enhances antibody-dependent cellular cytotoxicity [53]. Lapatinib, the initial tyrosine kinase inhibitor approved for BC treatment, triggers growth arrest and apoptosis in HER2-overexpressing cells by competitively inhibiting HER2 [54]. Neratinib is an orally-available small molecule tyrosine kinase inhibitor that effectively inhibits cell proliferation by irreversibly binding to HER1 and HER2, reducing their autophosphorylation and suppressing downstream signaling pathways [55].

## The structures and biology of KDM5 family

### The structures of KDM5 family

**Fig. 5:**
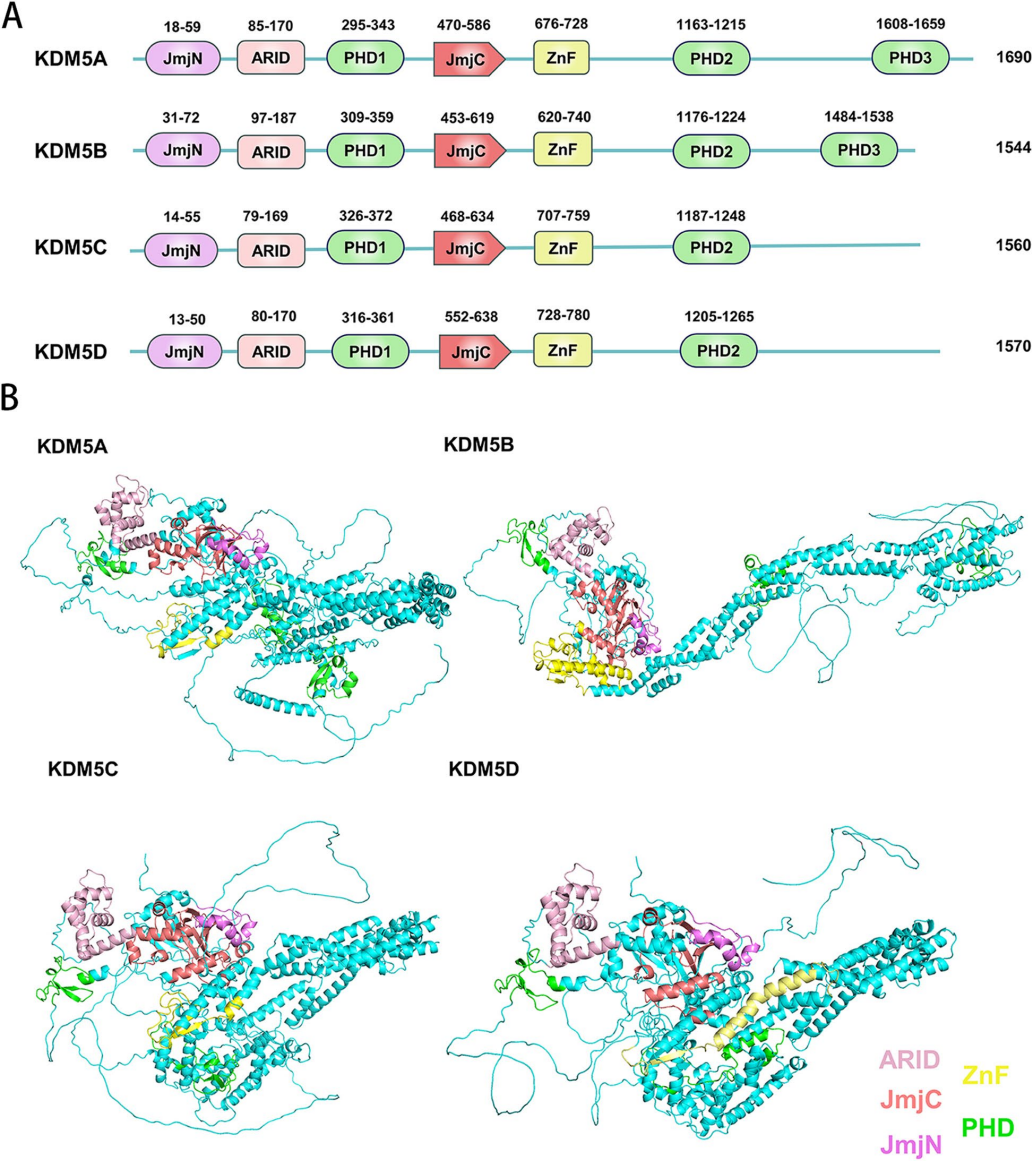
KDM5 structure. (**A**) KDM5 family members are 1560–1690 amino acid residues in length and contain six conserved structural domains including JmjN, ARID, PHD1, JmjC, ZnF, and PHD2. In addition, KDM5A and KDM5B have one more PHD3 structural domain than KDM5C and KDM5D. (**B**) The KDM5 family members are relatively similar in three-dimensional structure. The catalytic core structural domain JmjC is surrounded by ARID, JmjN, ZnF, and PHD1, which co-facilitate the demethylation reaction by binding to specific DNA sequences and target proteins

The members of the KDM5 family are highly conserved and possess five major structural domains, namely JmjC, JmjN, PHD, AT-rich interaction domain (ARID), and C5HC2 zinc finger (Fig. 5). Similar JARID2, ARID and PHD1 of KDM5 were inserted into the Jumonji domain, splitting it into JmjC and JmjN, but this domain arrangement is not common in other JmjC family members. The JmjC domain, originally defined by the amino acid similarity of KDM5A, KDM5C, and JARID2 [56], is a non-heme Fe(II) and 2-OG binding domain necessary for the JmjC proteins to carry out their catalytic reactions, in which the catalytic core is the conserved double-stranded β-helix fold (DBSH) [57]. Introducing a H499Y mutation at the binding site of Fe(II) eliminates the demethylase activity of KDM5B [58]. Furthermore, JmjN has a role in stabilizing the structure of KDM5 and its mutation reduces demethylase activity [58].

PHD is a common conserved structural domain in histone modifying enzymes that contains the key Cys4HisCys3 motif and zinc ion-coordinated residues, conferring KDM5 demethylation site-specificity by reading and binding histone-specific sequences [59]. KDM5A/B contain PHD1/2/3, while KDM5C/D contain only PHD1/2. PHD1 has a strong affinity towards unmethylated H3K4 (H3K4me0), which is influenced by residues modifications in the histone tail, and mutations occurring at conserved sites of PHD1 binding to H3K4me0 can abrogate this interaction [60–62]. The binding of PHD1 to the demethylation product H3K4me0 induces allosteric regulation of KDM5A conformation and establishes a positive feedback cycle between the “reader” and the “eraser” domain, thus further enhancing the demethylation activity of KDM5A [63, 64]. In addition, PHD1 of KDM5C can recognize histone inhibition mark H3K9me3 [65]. PHD3 in KDM5A and KDM5B tends to bind H3K4me3, contributing to the localization of the catalytic domains [60].

Compared to full-length KDM5A, ARID-deficient KDM5A exhibits reduced demethylation capacity of H3K4me3 possibly because this mutation alters spatial arrangements or global folding [66]. Molecular dynamics simulations reveal that the JmjC domain of ARID-deficient KDM5B is more flexible and can induce protein structure dynamic changes compared to full-length KDM5B [67]. Additionally, KDM5A achieves transcriptional regulation of specific genes through the binding of ARID to the CCGCCC motif [66]. In KDM5B, ARID selectively engages GCACA/C sequences to increase the selectivity of KDM5B for target genes. However, if this sequence is absent in the promoter, ARID can instead bind to target genes via the AATTAAA sequence [68]. Furthermore, the deletion of the entire ARID domain (residues 96–188) and a portion of JmjN (residues 69–73) generated by splicing of exons 2 and 4 leads to the abolishment of demethylation activity of KDM5B for H3K4me3 [67]. The deletion of PHD1 and ARID domains was found to have minimal impact on the enzymatic activity of KDM5C for demethylating H3K4me3 in vitro, while the C5HC2 zinc finger was essential for the KDM5 catalytic activity, possibly because it helped KDM5C to correctly bind to the substrate [69].

### The regulation of KDM5

**Fig. 6:**
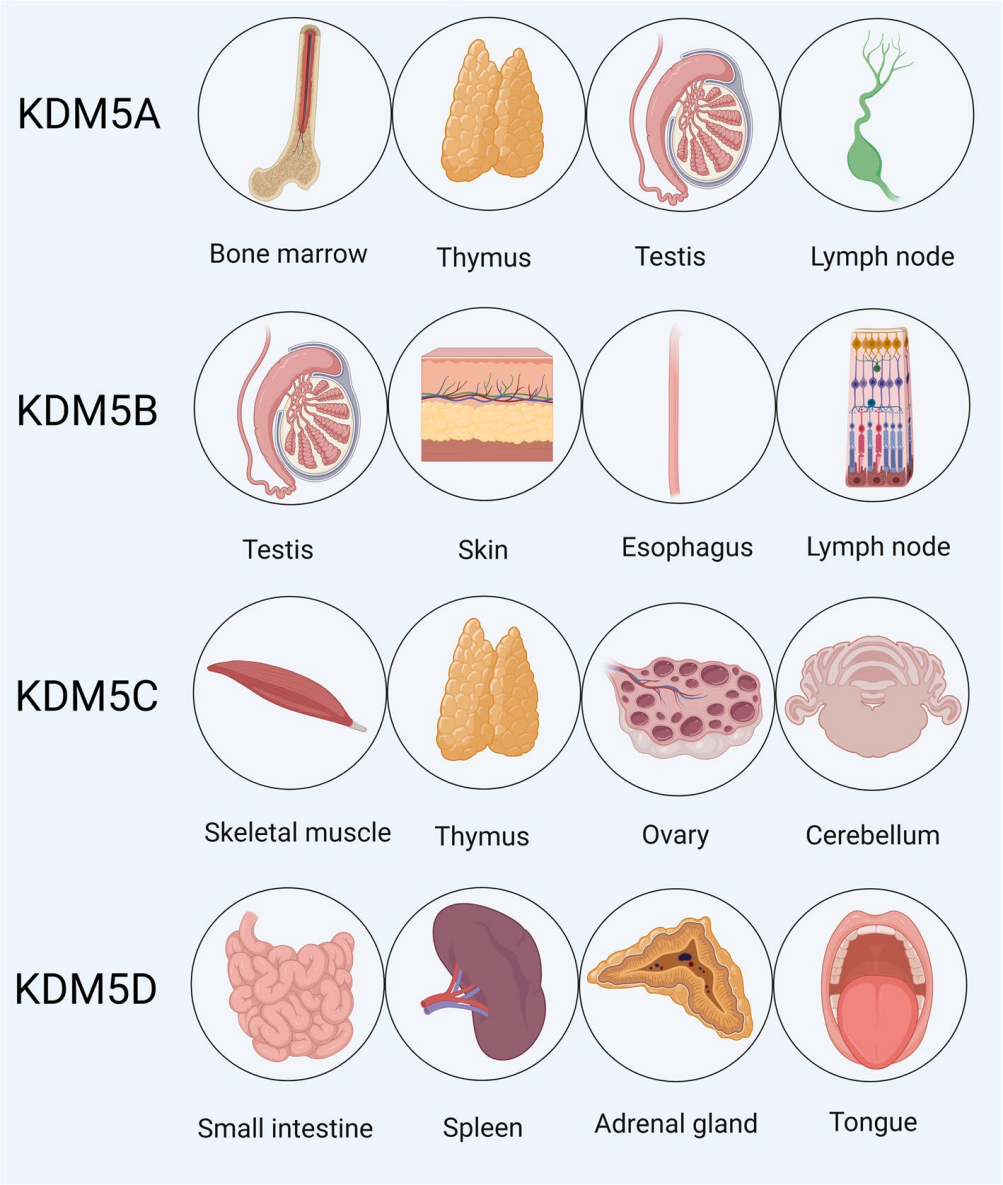
The four tissues with the highest expression of KDM5 proteins in humans. Data from the human protein atlas (https://www.proteinatlas.org)

Despite the similar structures of the KDM5 family, each isoform has different features and roles in both physiological and pathological environments. KDM5s are found in a range of human tissues, with the highest levels in the bone marrow, testis, skeletal muscle, and small intestine for KDM5A/B/C/D, respectively (Fig. 6). KDM5A and KDM5B have been indicated to be carcinogenic in numerous studies, while KDM5C and KDM5D may function as tumor suppressors, although the evidence is conflicting which may be due to the specific tumor microenvironment and different experimental conditions.

#### KDM5A

KDM5A, also named JARID1A or retinoblastoma-binding protein 2 (RBP2), was initially identified based on its capacity to interact with the retinoblastoma protein pocket domain [70]. KDM5A is involved in many physiological and pathological processes, including controlling cell proliferation, development, stemness, and mediating the epithelial-mesenchymal transition (EMT) by promoting or inhibiting transcription in a demethylase-dependent or demethylase-independent manner [71].

Upon DNA damage, KDM5A interacts with the RACK7-NuRD complex to repair double-strand breaks (DSB) by homologous recombination [72]. KDM5A drives a range of human cancers including acute myeloid leukemia, glioblastoma, renal cell carcinoma, and prostate, lung, gastric, and breast cancers [71]. Moreover, KDM5A promotes the differentiation of adipocytes through the C/EBPβ/KDM5A/Wnt 6 axis [73].

#### KDM5B

KDM5B, also known as JARID1B or PLU1, was initially identified in BC as an upregulated gene. KDM5B was required for the differentiation of mouse embryonic stem cells [74]. Embryos lacking KDM5B showed neonatal death mainly due to respiratory failure and defects in bone and neuron development [75]. However, KDM5B is upregulated in various cancers, and possibly through its effects on the level and distribution of H3K4me3 near the promoters of cancer-related genes. Phenotypically, KDM5B promotes cancer stem cells, DNA repair, EMT, and intratumoral heterogeneity [76]. A recent study has shown that KDM5B is essential for the complete activation of the NF-κB signaling cascade in macrophages and the secretion of proinflammatory cytokines, while inhibition of KDM5B could protect mice from immune injury [77].

#### KDM5C

Although KDM5C (JARID1C or SMCX) is located on the X chromosome, KDM5C can be expressed by evading X chromosome inactivation, and the KDM5C gene region lacks DNA methylation modification and H3K27me3 enrichment [78]. KDM5C is associated with various sex-dependent conditions, including autism, adiposity, X-linked intellectual disability (XLID), and osteoporosis [65, 77, 79, 80]. KDM5C was considered a tumor suppressor in different cancers such as BC, clear cell renal carcinoma, and cervical cancer by regulating enhancer function [81, 82]. However, other studies have suggested its oncogenic functions in other types of cancer [80, 83]. Therefore, clarifying its molecular mechanisms of action in specific environments is crucial.

#### KDM5D

KDM5D (JARID1D or SMCY) is found on the Y chromosome and may be involved in spermatogenesis [84]. Studies have shown that low levels of KDM5D in gastric, lung, and colorectal cancer is associated with poor prognosis [85–87]. Further findings indicate that KDM5D is a tumor suppressor inhibiting the division, invasion, and EMT of cancer cells. In addition, KDM5D also slows the development of prostate cancer by inhibiting the transcription of androgen target genes and metastasis-related genes via removing H3K4me3 marks [88, 89].

## KDM5 proteins in breast cancer

### KDM5A

KDM5A is increased at both mRNA and protein levels in BC cells [90]. Knockdown of KDM5A by shRNA significantly inhibited the normal and non-anchored growth of *KDM5A* amplified cells such as ZR-75-1, HCC1937, and SUM-149, but did not significantly affect the growth of non-KDM5A amplified cells SUM102 and normal mammary epithelial cells MCF-10 A [90]. EMSY has oncogenic effects in various cancers, including BC [91]. Immunohistochemistry showed significant co-regulation of the EMSY/KDM5A complex in the EMSY-positive BC subpopulation [92]. Further investigations revealed that KDM5A, with EMSY and SIN3B (the histone deacetylase complex subunit), forms a transcriptional complex that is then recruited by ZNF131 to the transcription start site (TSS) enriched for H3K4me3 to enhance transcription of target genes and stimulate cell proliferation. However, this biological mechanism has not been fully elucidated and may be due to a combination of epigenetic mechanisms such as acetylation, methylation, and deacetylation. The combination of a KDM5A inhibitor and deacetylase inhibitor has been hypothesized to be effective at both inhibiting cell proliferation by counteracting the overexpression of EMSY/KDM5A/SIN3B and, at the same time, preventing cancer cell resistance to drugs [71, 92].

Post-translational modifications (PTMs) of KDMs, such as phosphorylation, methylation, and ubiquitination, have significant effects on their function. These modifications can alter KDMs subcellular localization, stability, enzymatic activity, and interactions with other proteins, thereby impacting their role in BC and other diseases. The PI3K/AKT signaling axis is often hyperactive in BC and contributes to cancer progression [93]. Inhibiting the PI3K/AKT signaling pathway decreased H3K4me3 levels in various BC cells and lowered the expression of cell cycle-promoting genes [94]. Further investigation revealed that KDM5A is a target of AKT, and phosphorylation of KDM5A by AKT increases its localization in the cytoplasm while reducing its binding to chromatin, thereby enhancing the enrichment of H3K4me3 at TSS [94]. As the phosphorylation status of KDM5A determines its subcellular localization, this suggests that regulating the PTM of KDM5A may be a promising anticancer strategy.

Hypoxia is frequently observed in the tumor microenvironment [95]. EGLN2, an upstream oxygen-sensing factor, has been shown to specifically recognize H3P16 and catalyze proline hydroxylation forming H3P16OH, which contributes to the engagement of KDM5A (PHD3) to H3K4me3 to promote its demethylation [96]. Exposure to hypoxic conditions, decreasing levels of EGLN2, or treatment with a pan-proline hydroxylase inhibitor all led to decreased binding of KDM5A to H3K4me3, thereby increasing H3K4me3 levels. In addition, EGLN2 deficiency induced the expression of DDK1 (a Wnt signaling inhibitor) and decreased the proliferation of MDA-MB-231 cells, but had no effect on 293T cells. Additionally, a lowering of H3P16OH levels and an increase of H3K4me3 were observed in normal mammary tissues of *EGLN2*^*−*^*/*^*−*^ mice compared to the wild-type [96], suggesting that the EGLN2-H3P16OH-KDM5A axis is also involved in the regulation of H3K4me3 levels under physiological conditions.

Fbxo22 is a ubiquitin ligase that inhibits BC progression, and its expression has been correlated with longer survival in BC [97]. Fbxo22 enhances *P16* expression by upregulating H3K4me3 on the *P16* promoter through promoting KDM5A protein ubiquitination and degradation [98]. Overexpression of Fbxo22 resulted in DNA damage in TNBC cells as indicated by a significant increase in γH2AX, and effectively slowed tumor invasiveness and metastasis both in vitro and in vivo, however, it was reversed upon simultaneous overexpression of Fbxo22 and KDM5A [98].

In BC, the 3’-untranslated regions (3’-UTRs) of oncogene mRNA are commonly shortened, resulting in the absence of binding sites for mRNA degradation or translational repression, which leads to overexpression of oncoproteins promoting cancer progression [99]. In addition to the demethylation function, KDM5A has also been discovered to modulate the size of the 3’-UTR of mRNA. The yeast KDM5 protein JHD2 regulates mRNA 3’-UTR length by interacting with chromatin, mRNAs, and transcription factors in various ways including demethylation [100]. Moreover, KDM5A regulates *DICER1* 3’-UTR length either by demethylation or independently of demethylation [100]. Hence, when developing inhibitors to target KDM5A, it is essential to not only focus on its demethylation activity but also comprehensively evaluate the involvement of KDM5 in other factors contributing to disease progression.

Despite the effectiveness of chemotherapy in eliminating tumor cells, the development of drug resistance remains a significant challenge in chemotherapy. BC cells with amplified *KDM5A* were more prone to develop resistance towards the EGFR inhibitor erlotinib and with further upregulation of KDM5A expression in resistant cells [90]. KDM5A knockdown reduced the number of drug-resistant cells and promoted the expression of *P21* and *BAK1*, implying that KDM5A may induce cell resistance by regulating *P21* and *BAK1*. In addition, the knockdown of KDM5A or treatment with KDM5 inhibitors, such as KDM5-C49 or KDM5-C70, augmented the sensitivity of endocrine-resistant luminal BC cells to fulvestrant [101]. Furthermore, the combination of fulvestrant and KDM5 inhibitor substantially enhanced apoptosis and reduced tumor volume compared to individual treatments in vivo. Mechanistically, KDM5 inhibitor treatment reduced transcriptome heterogeneity in luminal ER + BC cells and endocrine-resistant cells. In addition, KDM5 inhibitor-resistant cells showed heightened levels of H3K27me3, whereas treatment with the E2H2 inhibitor GSK126 lowered H3K27me3 levels and increased sensitivity to KDM5 inhibitor, suggesting that KDM5 inhibitor resistance is acquired resulting from modified epigenetic mechanisms and is distinct from innate fulvestrant and tamoxifen resistance [101].

However, several studies have indicated that KDM5A may have a positive function in suppressing BC. In the GOBO database, *KDM5A* was less expressed in different types of BC with poorer clinical outcomes [102]. Furthermore, in BC patients treated with docetaxel, *KDM5A* was higher in tumors that exhibited a better pathologic complete response rate [102]. Additionally, treating MCF-7 cells with the ginsenoside Rg3 inhibited cell proliferation and induced apoptosis, and also led to a decrease in *KDM5A* CpG methylation levels thereby increasing KDM5A expression. In contrast, the knockdown of KDM5A mitigated the inhibitory effect of Rg3 on MCF-7 cells [103].

### KDM5B

Female mice with knockout of *KDM5B* exhibited retarded mammary gland development accompanied by lowered blood estrogen levels and decreased mammary epithelial cell division [104]. In contrast, KDM5B promotes pubertal mammary duct growth by regulating systemic estrogen levels and the transcription of important mammary development regulators including *FOXA1* and *Stat5a* [104]. Additionally, KDM5B is necessary for mouse embryo survival, and knockdown of KDM5B leads to early embryo death. Although mice with a deletion of the KDM5B ARID domain were viable, they also exhibited a delayed mammary development phenotype [105]. Moreover, KDM5B and HDAC4 co-expression in differentiated mouse mammary glands and breast carcinomas indicates that their interaction may be associated with transcriptional repression of KDM5B under both physiological and pathological conditions in these tissues [106]. Therefore, KDM5B has a crucial function in embryonic development and the regulation of normal mammary gland development. Conversely, KDM5B dysfunction may be one of the potential causes for the occurrence of BC.

KDM5B expression was higher in cancer cells than in adjacent normal cells in immunohistochemical analysis of tumor samples from 176 women with invasive ductal carcinoma [107]. Moreover, a positive association was observed between increasing tumor grade and KDM5B expression, while simultaneously, a negative correlation was identified between P16 and KDM5B expression [107]. Therefore, combined P16 gene therapy and KDM5B targeted therapy could a viable approach to combat BC mechanisms. Moreover, KMD5B is often upregulated in luminal breast cancers where it plays a critical role in regulating the expression of luminal cell-specific programs [108].

KDM5B typically binds to target gene promoters to decrease their transcription. For example, KDM5B inhibits *CAV1*, *HOXA5*, and *BRCA1* transcription by binding and decreasing the level of H3K4me3 on their promoter, and ultimately driving the progression of MCF-7 cells in G1 phase [58]. Knockdown of KDM5B reduces the division of 4T1 cells and decreases the proliferation of tumor cells in vivo [58]. CUT-like homeobox 2 (CUX2) is expressed highly in tumor relative to normal samples, and its knockdown decreases the growth and invasive ability of BC cells, whereas the opposite effect is observed for SOX17 [109, 110]. Further investigations revealed that CUX2 promoted KDM5B expression by recruitment to the *KDM5B* promoter, while KDM5B inhibited SOX17 expression in a demethylation-dependent manner [109]. Thus, inhibiting CUX2 or KDM5B by targeting the CUX2/KDM5B/SOX17 axis thereby increasing the level of SOX17 could potentially be a BC treatment strategy. The cell cycle inhibitor p21cip (CDKN1A) can mediate cell cycle blockade through both p53-dependent and non-dependent pathways [111]. Estrogen-responsive genes *TFAP2C* and *Myc* are overexpressed in BC with poor prognosis, and lack of regulation of these genes is linked to the absence of CDKN1A resulting in anti-estrogen therapy resistance [112, 113]. TFAP2C, Myc, and KDM5B combine to form a ternary complex near CDKN1A promoter to repress CDKN1A in MCF-7 cells, while pharmacological induction of CDKN1A resulted in a decrease of the TFAP2C-Myc-KDM5B complex, resulting in cellular arrest in the S or G2/M phase [114]. Hence, KDM5B exhibits synergistic interaction with the TFAP2C/Myc complex in BC, which effectively overcomes cell cycle arrest by inhibiting CDKN1A.

Mutations of transcription factor FOXP3 are linked with the pathogenesis of many cancers [115]. MOF is a MYST family histone acetyltransferase that selectively acetylates histone H4K16 [116]. In MCF-7 cells, FOXP3 recruits MOF to the binding site and induces H4K16 acetylation. Subsequently, either by competing for DNA binding or through other actions, FOXP3 stimulates KDM5B to translocate from the FOXP3 binding site, thus increasing H3K4me3 and facilitating transcription [44]. This study reveals the new facet of KDM5B in the transcription process. Hexamethylene bis-acetamide (HMBA)-inducible protein 1 (HEXIM1) is downregulated in a variety of cancers and negatively correlates with proliferative activity [117, 118]. HMBA and 4a1, which are HEXIM1 inducers, were found to promote *HEXIM1* expression by inhibiting KDM5B demethylation at the *HEXIM1* promoter. Molecular docking suggests that HMBA and 4a1 may occupy methylated lysine histone substrate binding sites [118]. This opens up new leads for the discovery of compounds targeting KDM5B for the treatment of BC.

KDM5B also remodels cancer cell metabolism to promote BC progression. By inhibiting the AMPK signaling pathway, KDM5B upregulates key genes that regulate lipid metabolism (including *FASN* and *ACLY*), thereby inducing lipid metabolic reprogramming and promoting the development of BC [119]. This suggests that targeted therapy against KDM5B is an effective strategy for managing abnormal lipid metabolism in BC.

BC brain metastasis (BCBM) is a type of distant metastasis that occurs in late-stage BC patients, with a low mean 1-year overall survival [120]. Analysis of two GEO datasets containing BCBM revealed that *ANLN*, *BUB1*, *TTK*, and *SKA3* are hub genes for the development of BCBM and predictive factors for poor survival in BC. Three transcription factors including KDM5B were identified as key regulatory factors for these four hub genes [121]. In addition, through bioinformatics analysis of BC gene expression profiles obtained from nine GEO databases, researchers identified KDM5B as a transcriptional regulator of key BC genes including *EGFR*, *FN1*, *EZH2*, *MET*, *CDK1*, *AURKA*, *TOP2A*, and *BIRC5* [122].

MicroRNAs (miRNAs) are small noncoding RNAs that modulate gene expression by interacting with the 3’-UTR of mRNA [123]. miR-381-3p and miR-486-5p were found to bind to the *KDM5B* mRNA 3’-UTR thus reducing KDM5B protein levels as well as promoting KDM5B target gene *BRCA1* mRNA levels [124]. Given that BRCA1 promotes G1/S arrest through p53-dependent or independent actions, its loss has a key function in BC cells evading cell cycle regulation [125], further discovered that overexpression of miR-381-3p or miR-486-5p leads to an increased G1/G0 phase arrest and enhanced sensitivity to radiation in MCF-7 cells [124]. This suggests that miR-381-3p and miR-486-5p can interfere with KDM5B-mediated DNA damage repair by inhibiting KDM5B. Additionally, miR-137 decreases the growth and migration of MCF-7 cells by binding to *KDM5B* mRNA 3’-UTR [126]. Furthermore, KDM5B promotes MCF-7 cell cycle progression by increasing the expression of *cyclin D1* via inhibiting miRNA *let-7e* expression in a demethylase-dependent manner [127]. Research has found that KDM5B and ETS-1 jointly recruit EMSY to the coding anti-metastatic microRNA miR-31 promoter to reduce miR-31 expression, thus promoting invasive and migratory characteristics by inducing the transformation of BC cells [128]. Therefore, targeting of KDM5A and KDM5B may provide a new intervention approach for EMSY-driven BC. Additionally, hsa-miR-448 has been predicted to target the degradation of *KDM5B* mRNA, exerting a negative regulatory effect on the function of KDM5B in TNBC [129]. Furthermore, the activity of the long non-coding RNA (lncRNA) MALAT1 induced by KDM5B amplifies the transcription of metastasis-related targets *snail* and *vimentin*, thereby promoting EMT activation and facilitating the migration of TNBC cells [129]. Moreover, increased levels of KDM5B are correlated with the shortening of the 3’-UTR of oncogene *CCND1*. Treating MCF-7 cells with KDM5 inhibitor KDM5-C70 led to an overall rise of H3K4me3 levels but did not affect the length of *CCND1* 3’-UTR, suggesting that regulation of *CCND1* 3’-UTR length does not require the demethylation activity of KDM5B [100].

Evading immune surveillance is a major hallmark of cancer, and one common mechanism involves the inhibition of the stimulator of interferon genes (STING)-dependent innate immune response [130]. BC cells signal through the KDM5B-STING axis to evade the detrimental effects of innate immune responses induced by cytoplasmic DNA [131]. Specifically, STING was silenced by KDM5B via removing H3K4me3, thereby blocking cytoplasmic DNA-initiated signaling mediated by the cGAS-STING-TBK1-IRF3 axis. Moreover, inhibition or depletion of KDM5B enhances STING expression and activates IFN-stimulated genes (ISGs). Thus, KDM5B may function as a promising target for cancer immunotherapy and the combination of KDM5 inhibitors and STING agonists could maximize the antitumor immune response. Multimer staining of circulating T cells from BC patients revealed a higher population of multimer positive CD8 + T cells for two of three JARID1B epitopes tested compared to healthy adults [132]. Furthermore, in vitro, KDM5B protein induced IFN-γ production in stimulated CD8 + T cells [132]. Given the oncogenic role of KDM5B in BC, using KDM5B as an antigen to stimulate CD8 + T cells and induce cytotoxicity against cancer cells is a potential antitumor immunotherapy approach.

Recent research has identified a truncated and catalytically inactive isoform of KDM5B, named KDM5B-NTT, which lacks the entire JmjN domain and part of the ARID domain and is more stable compared to the full-length KDM5B [133]. In MCF-7 cells, overexpression of KDM5B-NTT increased H3K4 methylation and derepresses the tumor suppressor Cav1 and several other genes in the IFN-α and IFN-β response [133]. Thus, the correlation between KDM5B isoforms and their regulation in BC should be further investigated. Furthermore, KDM5B is closely associated with increased transcriptional heterogeneity and has a promoting role in promoting chemoresistance, particularly in luminal subtype BC cells [101].

KDM5B was also found to be associated with the inhibition of BC progression. The chemokine CCL14, which correlates with the angiogenic and metastatic capabilities of BC cells, is negatively regulated by KDM5B [134]. Specifically, the KDM5B/LSD1/NuRD complex binds to the CCL14 promoter and suppresses its transcription by reducing H3K4 methylation levels, thus effectively inhibiting the invasive ability of BC cells and angiogenesis in vivo. This study suggests that KDM5B can act as an anti-oncogenic factor by synergizing with HDM and HDAC to manipulate chemokine networks. KDM5B (Ser1456) phosphorylation catalyzed by CDK1 inhibited tumor stemness genes *SOX2* and *NANOG* expression by reducing the enrichment of KDM5B at their promoters [135]. In addition, KDM5B phosphorylation required HEXIM1 and is cell cycle-dependent, with KDM5B phosphorylation at the highest levels during the G2/M phase [135]. Moreover, disruption of the SIN3A-PF1 interaction suppressed TNBC growth, migration, and invasion by inhibiting the expression of ITGA6 and ITGB1 through increasing SIN3A/KLF9/HDAC2 and KDM5B recruitment near their promoter [136].

### KDM5C and KDM5D

TRIM11 is an E3 ubiquitin ligase that contains a RING finger domain, and its expression has been linked with cancer [137]. Upregulation of TRIM11 enhances the growth and migration abilities of MDA-MB-231 cells, and also promotes tumor growth in vivo, while KDM5C activity inhibits tumor progression and rescues the phenotype caused by TRIM11 [138]. Mechanistically, TRIM11 upregulates MCAM (a pro-tumorigenic factor) and downregulates the expression of immune-related genes by facilitating KDM5A (K48-linked ubiquitin chain) proteasome degradation [138].

Furthermore, multiple studies have suggested that the interplay between KDM5C and receptor for activated C-kinase 7 (RACK7) inhibits BC [139, 140]. Although adriamycin effectively kills cancer cells, it has been observed that lower doses can lead to increased chemoresistance, migration, and stemness. However, overexpression of RACK7 was shown to reverse sublethal adriamycin-promoted resistance in vitro and in vivo [139]. In terms of mechanism, RACK7 forms a transcriptional repressor complex with KDM5C and EZH2 to maintain high levels of H3K27me3 and low levels of H3K4me3 in the promoters of genes for EMT, drug-resistance, and stemness, ultimately induce sensitivity to chemotherapy [139]. Moreover, a significant overlap between the enhancer sites occupied by KDM5C and RACK7 was observed [140]. Further studies revealed that RACK7 recruits KDM5C to enhancers site and reduces H3K4me3 levels, thereby repressing the transcription of several oncogenes including *S100A*. When KDM5C or RACK7 was absent, it led to enhanced invasion and migration of ZR-75-30 cells and promotion of tumor growth in vivo through de-repression of oncogenes [140]. This research indicates that RACK7 and KDM5C may play a role in inhibiting the occurrence of cancer.

Conversely, KDM5C may have a function in promoting breast tumorigenesis by directly activating ER𝛼-target genes and indirectly repressing IFNs and ISGs to evade immune surveillance [141]. Specifically, KDM5C interacts with RACK7 and is recruited by ERα to the enhancers of ER𝛼-target genes, where they interact with CDK9 and CCNT1 in the P-TEFb complex to upregulate ER𝛼-target expression. Notably, upon binding to KDM5C, ERα masks its demethylase activity. In addition, KDM5C decreases TBK1 phosphorylation and thus suppresses IFN and ISG expression [141]. This finding suggests that KDM5C may play an oncogenic role in ER + BC through a dual mechanism involving transcriptional activation and inhibition, which is contingent upon specific environmental and enzymatic activity requirements.

KDM5C also promoted MDA-MB-231 and BT549 cell migration and invasion by inhibiting the expression of BC metastasis suppressor 1 (BRMS1) in a demethylase-dependent manner [142]. Moreover, miR-138 effectively inhibited the proliferation of MCF-7 cells by directly engaging 3’-UTR of *KDM5C* [124].

The KDM5D coding gene is situated on the Y chromosome. Although male BC is a rare disease, its incidence has been increasing annually [143]. Considering the inhibitory role of KDM5D in various cancers, exploring the mechanism of KDM5D in male BC may become an academically valuable and attractive topic.

## KDM5 inhibitors

According to previous studies, KDM5A/B/C are associated with BC, either in pro-tumorigenic or anti-cancer roles. The role of KDM5 in BC is complex, and many of the mechanisms proposed to date remain incomplete. Nevertheless, this does not impede the enthusiasm of the scientific community for investigating KDM5 inhibitors in BC, as it contributes to the development of KDM5 inhibitors and also facilitates elucidating in the role of KDM5 in BC (Table 2).

**Table 2:** Preclinical KDM5 inhibitors in breast cancer

Compounds	Structure or Names	Category	Cell lines, EC_50_	IC_50_	Other Cancers (EC_50_)	Refs
**1**		Metal-based complexes	MDA-MB-231 = 90.1 nM 4T1 = 68.2 nM MCF-7 = 0.27 µM MCF-10 A = 1.0 µM HEK293T = 3.6 µM LO2 = 4.7 µM	KDM5A = 23.2 nM	-	145
**2**		Natural product	MDA-MB-231 = 0.54 µM MDA-MB-468 = 0.71 µM MCF-7 = 2.26 µM MCF-10 A = 8.21 µM LO2 > 10 µM	KDM5A = 23.8 nM KDM4A = 100 µM	-	146
**3**		Pyrimidinones	MCF-7 > 31.6 µM T-47D > 25 µM EFM-19 > 15 µM	KDM5A = 10 nM KDM5B = 3 nM KDM5C = 14 nM KDM2B > 25 µM KDM3B > 25 µM KDM4C = 2 µM	PC9 > 25 µM, Hs888 > 25 µM, Colo205 > 25 µM	147, 148
**4**		Thiotriazoles	-	KDM5A = 1.32 µM KDM5B = 50 µM KDM5C = 6.56 µM KDM6A > 50 µM KDM6B > 50 µM	-	149
**5**		Thiotriazoles	ZR-75-1 < 100µM, MDA-MB-231 > 100 µM	KDM5A = 2.66 µM KDM5B = 50 µM KDM5C = 7.12 µM KDM6A > 50 µM KDM6B > 50 µM	Hela > 100 µM	149
**6**		Pyrazolylpyridine derivative	ZR-75-1 = 90 nM	KDM5A = 13 nM KDM5B = 2 nM KDM4C = 41 nM KDM6B = 2.3 µM KDM1A > 40 µM KDM2A = 0.82 µM KDM2B = 0.63 µM	-	150, 151
**7**	KDM5-Inh1 patent Number: WO 2014/131,777 A1	-	BT-474 < 0.1 µM, SK-BR-3 < 1 µM	KDM5A = 4.3 nM KDM5B = 0.28 nM KDM5C = 6.4 nM KDM4A = 7.6 nM KDM4B = 6.4 nM KDM4C = 6.0 nM KDM6A = 6200 nM KDM6B = 6200 nM KDM7B = 400 nM	-	122
**8**		Isothiazolones	UACC-812 < 10 µM	KDM5A = 6.01 µM KDM5B = 4.08 µM KDM5C = 4.92 µM	-	152
**9**		Aminomethylpyridines	MDA-MB-231 < 1 µM BT549 < 5 µM MDA-MB-468 < 5 µM MDA-MB-453 > 10 µM	KDM5A = 71 nM KDM5B = 19 nM KDM5C = 69 nM KDM5D = 69 nM KDM2B = 4400 nM KDM4C = 4800 nM	HeLa = 50 µM MM1S = 30 µM	118, 153 154
**10**		Thioamides	MCF-7 > 3 µM	KDM5B = 2.5 µM KDM5C = 2 µM	HeLa < 30 µM	154, 155
**11**		Thioamides	MCF-7 > 3 µM	KDM5 = 1 µM	-	154
**12**		2,4-PDCA analog	-	KDM5A = 7 nM KDM5B = 4 nM KDM5C = 13 nM KDM5D = 15 nM	-	101, 156
**13**		2,4-PDCA analog	MCF-7 = 5 µM	-	MM.1 S = 20 µM	101, 141, 156
**14**		Pyrimidinones	-	KDM5A = 15.1 nM	PC9 = 340 nM	101, 157

-: No data

KDM5A and KDM5B primarily function as oncogenes and are associated with the progression of BC, making them suitable targets for BC. Metal complexes diverse a variety of metal oxidation states and geometries for the arrangement of ligands, allowing them to form shape-specific interactions with target proteins [144]. Our previous study has found that the rhodium(III) complex (**1**) (carrying two 2-phenylquinoline C^N ligands and a 4,4’-diphenyl-2,2’-bipyridine N^N ligand) exhibited high selectivity for KDM5A with half-inhibitory concentration (IC_50_) value of 23.2 ± 1.8 nM compared to other histone demethylases including KDM1A, KDM4A and KDM6A [145]. Both **1** treatment or knockdown of KDM5A significantly reduced H3K4me2/3 levels in MDA-MB-231 cells, while KDM5A knockdown reduced the effect of **1** to cells, suggesting that **1** exerts its pharmacological actions by directly engaging KDM5A. Mechanistically, **1** impaired the binding between KDM5A and H3K4me2/3, resulting in the buildup of H3K4me2/3 at the promoter of the tumor suppressor p27 and an increase of *p27* expression. This resulted in antiproliferative activity and induction of cell cycle arrest in a variety of BC cell lines while exhibiting low cytotoxicity to normal cells. Furthermore, **1** inhibited tumor growth in TNBC model mice in a dose-dependent manner and exhibited lower organ toxicity compared to cisplatin and adriamycin.

In addition, our research group used high-throughput virtual screening to identify a potential compound (**2**) targeting KDM5A [146]. Molecular docking analysis revealed that compound **2** occupied a region that is normally occupied by 2-OG, with predicted a hydrogen bond interaction with N493. **2** promoted BC cell cycle arrest and cell senescence by upregulating *P16* and *P27* via inhibiting the interaction of KDM5A with H3K4me3 at their promoters. In terms of cytotoxicity, **2** was less toxic to the normal LO2 and MCF-10 A cells compared to BC MDA-MB-231 (0.54 µM), MDA-MB-468 (0.71 µM) and MCF-7 cells (2.26 µM).

Compound CPI-455 (**3**) was modified from an inhibitor of KDM4C, the most similar enzyme to the KDM5 family [147]. **3** exhibited an IC_50_ value of 10 ± 1 nM against KDM5A and it had similar inhibitory activity against KDM5B/C, and was also 200-fold more selective than KDM4C. The X-ray structure of the KDM5A_12–797_-**3** complex revealed a single interaction between the nitrile group of **3** and the metal ion, while a hydrogen bonding interaction was also observed between the carbonyl oxygen of **3** and N575. Additionally, the central aromatic core of **3** engaged in π-π stacking with the aromatic residues Y472 and F480 and also formed edge-face aromatic contacts with W503. In vitro experiments, **3** increased H3K4me2/3 in a concentration-dependent fashion in M14 (melanoma), SKBR3 (BC), and PC9 (non-small cell lung cancer) cells. Although **3** did not significantly affect the growth or survival of parental cells, it decreased the number of lapatinib-resistant SKBR3 and PI3 kinase inhibitor-resistant EVSA-T cells. This result further suggests that the demethylating function of KDM5 is important for the survival of drug-resistant cancers. Furthermore, the combination of **3** with the DNA methyltransferase inhibitor decitabine not only enhanced the upregulation of H3K4me2/3 but also promoted decitabine-induced transcriptional activation of genes involved in homeostasis and immune regulation. In addition, this drug combination significantly decreased the viability of MCF-7, T-47D, and EFM-19 cells [148]. Future studies of KDM5 antagonists should explore whether this synergy can be further improved, as well as apply to these drug combinations in animal studies.

Using AlphaScreen technology (PerkinElmer), researchers identified compounds **4** and **5** as inhibitors of KDM5A demethylation in vitro [149]. Further investigation revealed that only **5** exhibited cell permeability and inhibited the proliferation of ZR-75-1 and MBA-MD-231 cells, while not affecting the growth of MCF-10 A cells. Treatment with **5** alone did not affect the growth of BT474 cells, but significantly reduced colony formation when co-treated with a low dose of trastuzumab (5 µg/ml).

4’-((2-Aminoethyl)carbamoyl)-[2,2’-bipyridine]-4-carboxylic acid is a potent 2-OG KDM4E competitive inhibitor. It interacts using its pyridyl nitrogens with the cofactor Fe(II) of KDM4E [150]. From this, a library of pyrazolylpyridine core-containing molecules was designed to achieve inhibition of KDM5 activity with high selectivity in the KDM family, among which compound **6** exhibited the best inhibition against KDM5A (IC_50_ = 13 nM) and KDM5B (IC_50_ = 2 nM) [151]. **6** significantly increased the level of H3K4me3 even at extremely low concentrations in ZR-75-1 cells. Additionally, orally administered **6** demonstrated excellent kinetic solubility, and it sustained high levels of H3K4me3 for 24 h in xenograft BC tissue following 5 days of treatment. However, there is a shortage of experimental data about the inhibitory potency of **6** on tumors in both in vitro and in vivo.

As a pan-KDM5 inhibitor, KDM5-inh1 (**7**) effectively suppresses the proliferation of HER-positive BC cell lines and induces apoptosis [122]. **7** exhibits a synergistic effect with trastuzumab or lapatinib in HER2 + cells, via downstream regulation of HER2 and AKT signaling axes. Moreover, the administration of **7** led to a reduction in tumor volume with low toxicity to mice. This result suggests that KDM5 inhibitors can supplement HER + BC treatment therapies to prevent the development of drug resistance.

Through high-throughput screening with the AlphaScreen platform of over 15,000 molecules, several compounds were identified as inhibitors of KDM5B. Among these compounds were known JmjC protein inhibitors such as 2,4-pyridinedicarboxylic acid (2,4-PDCA, IC_50_ = 4.3 µM for KDM5B, 4.1 µM for KDM5A) and catechols, along with a newly discovered inhibitor of KDM5B, 2–4(4-methyl phenyl)-1,2-benzothiazole-3(2 H)-one (PBIT, **8**) [152]. PBIT had inhibitory activity against KDM5A/B/C with respectively IC_50_ values of 6.01, 4.08, and 4.92 µM in vitro. In addition, PBIT significantly reduced H3K4me3 in Hela, MCF-7, and MCF10-A cells, and also killed most of UACC-812 cells at a concentration of 10 µM, however the toxicity to MCF-7 and MCF-10 A was minimal.

KDOAM25 (**9**) is a KDM5 2-OG competitive inhibitor, with an IC_50_ value < 100 nM for KDM5A/B/C/D in vitro, and no inhibition of other 2-OG oxygenases below 4.8 µM. The carboxamide of KDOAM25 forms an inverse hydrogen bonding interaction with residue Y425 in the carboxylate-binding pocket of KDM5B, which potentially accounts for the selectivity towards KDM5 [153]. In addition, **9** induced cell cycle arrest in myeloma cells and increased the sensitivity of MCF-7 cells to irradiation [153, 154]. **9** inhibited the proliferation of TNBC cells, induced cell differentiation, and increased the sensitivity of MDA-MB-453 cells to adriamycin. In vivo, **9** reduced tumor mass and lung metastasis and without inducing adverse effects including weight decrease or thrombocytopenia in mice [118].

RS3195 (**10**), a 2-OG competitive inhibitor, specifically inhibits the activity of KDM5B and KDM5D in vitro [155]. However, **10** induced only a modest rise of H3K4me3 levels in MCF-7. Moreover, the aryl hydrocarbon receptor response pathway was upregulated in **10**-treated MCF-7 cells, possibly due to its potential mechanism of toxicity [154]. Substitution of the phenyl ring of **10** to the pyrrole nucleus yielded RS5033 (**11**), which exhibited better upregulation of H3K4me3 but without affecting the cell cycle. In addition, **11** made MCF-7 cells more sensitive to irradiation and increased the accumulation of DNA damage [154]. This study confirms that chemical inhibitors targeting KDM5B can sensitive BC cells to radiation-induced damage.

KDM5-C49 (**12**), an analog of 2,4-PDCA, has been shown to inhibit the KDM5 enzyme activity at nanomolar concentrations by binding to the 2-OG binding site, with the pyridine and the aminomethyl nitrogen atoms interacting in a bidentate fashion catalytic Fe(II) ion [156]. To improve cell permeability, researchers have designed the ethyl ester derivative KDM5-C70 (**13**), which has been shown to reduce transcriptional heterogeneity in multiple BC cell lines [101, 156]. Furthermore, the combination of the ERα antagonist fluvastatin with KDM5-C70 had a synergistic effect on inhibitory cell proliferation of MCF-7, T47D, and BT474 cells [141]. In addition, combination therapy with C48 (**14**) and fulvestrant significantly reduced tumor volume but neither drug alone had the same effect [101, 157].

## Conclusion

**Fig. 7:**
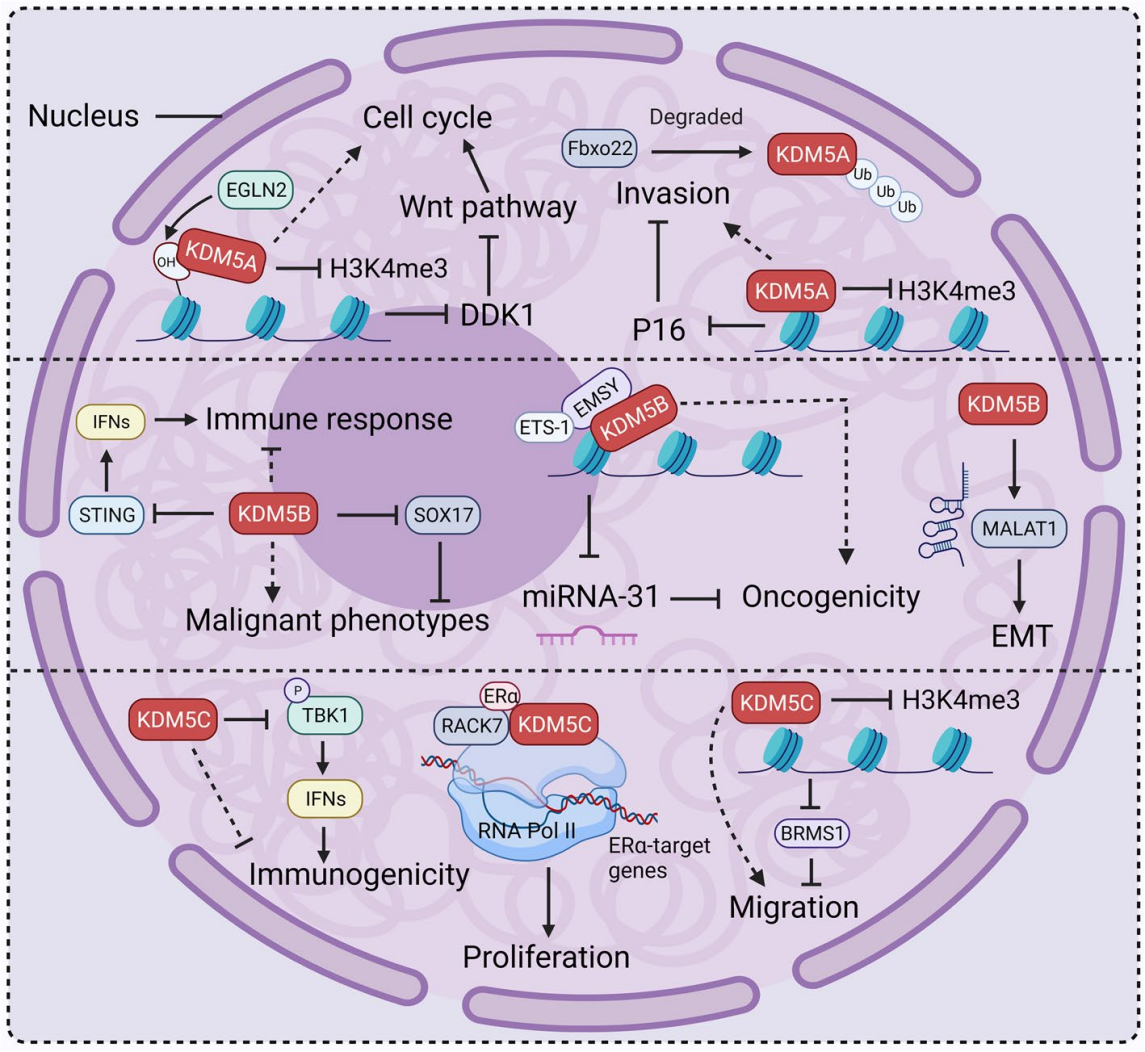
KDM5 proteins drive breast cancer progression. EGLN2-catalyzed hydroxylation of H3P13 promotes the binding of KDM5A to H3K4me3 thereby inhibiting the expression of the Wnt signaling inhibitor DDK1 and indirectly promoting Wnt signaling-induced cell cycle. KDM5A also suppresses the expression of the tumor suppressor gene P16 thereby promoting the migration of breast cancer cells. KDM5B impairs the immune response by inhibiting STING and promoting cancer cell migration by inhibiting SOX17. KDM5B interacts with EMSY to inhibit miRNA-31 thereby promoting tumorigenesis. In addition, KDM5B enhances cancer cell EMT by promoting MALAT1. KDM5C inhibits the expression of immune genes to promote tumorigenesis, and it interacts with RACK7 to promote the expression of ER target genes, which promotes cancer cell proliferation. KDM5C inhibits the expression of BRMS1 in a demethylation-dependent manner to promote cell migration

**Fig. 8:**
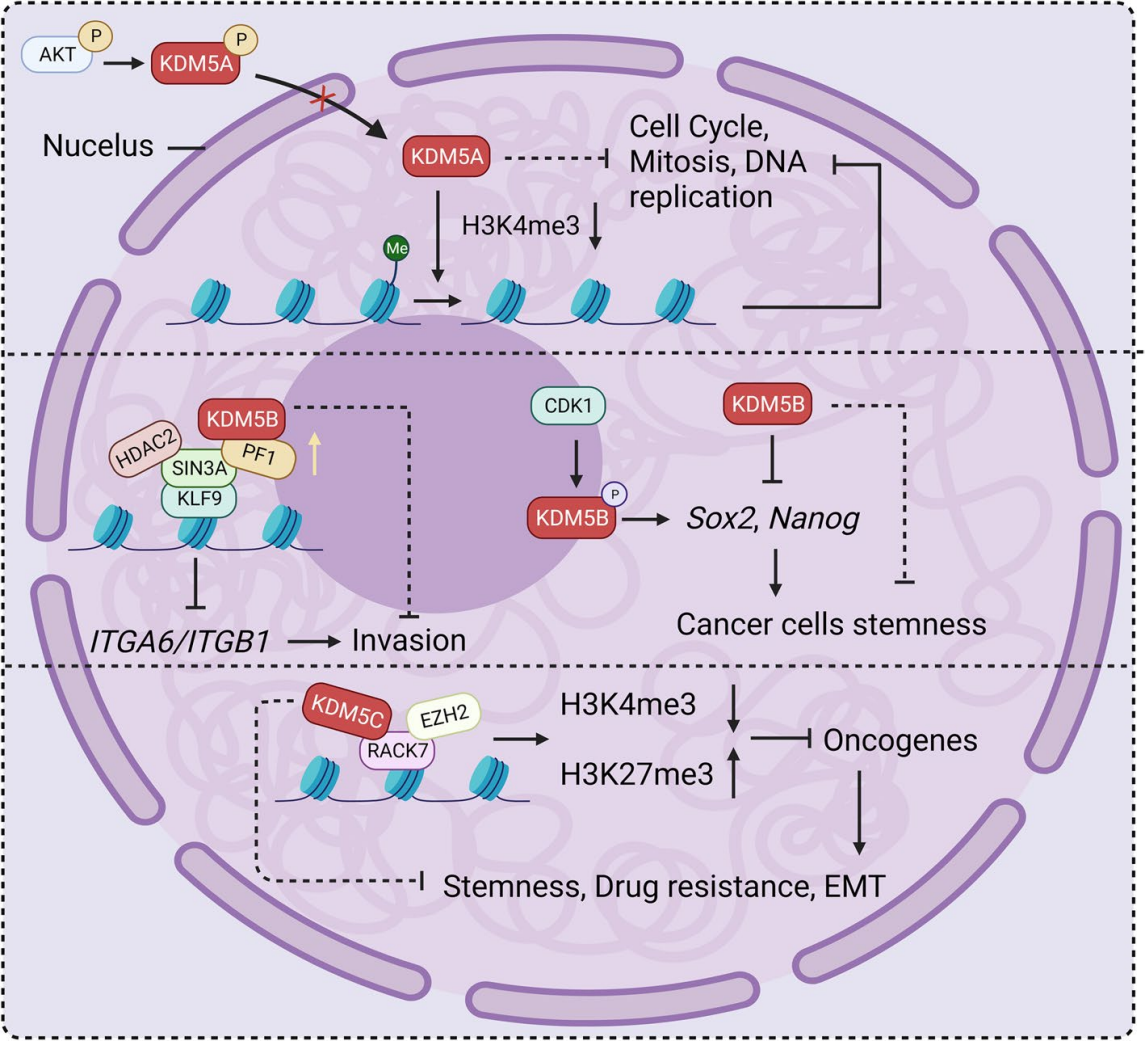
Some studies find that KDM5 proteins may inhibit breast cancer progression. AKT-catalyzed phosphorylation of KDM5A restricts the access of KDM5A to the nucleus, and KDM5A in the nucleus inhibits the expression of proteins related to the cell cycle, mitosis, and DNA replication by decreasing methylation on TSS H3K4. KDM5B localizes to the promoters of ITGA6 and ITGB1 along with the SIN3A complex to repress their expression thereby inhibiting breast cancer invasion. Moreover, KDM5B promotes the stemness phenotype of breast cancer cells by suppressing the expression of the cell stemness genes Sox2 and Nanog. RACK7 localizes to oncogenes with EZH2 and KDM5C to inhibit their expression by regulating histone bivalent modifications thereby suppressing cell stemness, drug resistance, and EMT

**Fig. 9:**
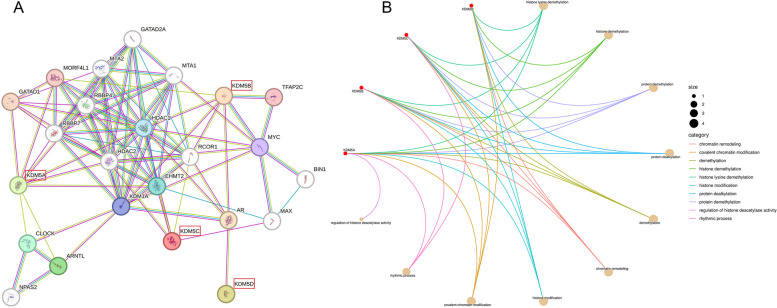
(**A**) The protein-protein interaction network of KDM5 proteins as predicated by STRING (https://string-db.org). (**B**) Biological function analysis of the KDM5 proteins as predicated by Bioinformatics (http://www.bioinformatics.com.cn)

Epigenetic modifications have a crucial function in BC and have raised high hopes for the use of epigenetic tools in diagnosing and treating BC. The KDM5 family are important regulators of H3K4me2/3 markers, and act either alone or together with other proteins to regulate transcription at the promoter or enhancer sites of target genes, thereby influencing cell cycle, migration, metabolism, and immune responses in BC (Figs. 7 and 8). Besides, many studies also have reported that KDM5s are involved in breast cancer progression in a demethylation-independent manner such as through PTMs or the regulation of 3-‘UTRs. Therefore, to comprehensively comprehend the roles of KDM5 in BC, it is imperative to investigate the unexplored mechanisms and elucidate their interrelationships. Protein-protein interaction (PPI) network analysis of KDM5 family members can provide important information for understanding their function in BC (Fig. 9).

As members of the KDM5 family share similar structural domains and same substrates, their functions can be considered somewhat redundant. However, merely focusing on one member within a particular context may prove inadequate in elucidating their contribution to BC. In contrast, a concurrent examination of two or more individuals can yield substantial benefits, allowing for a comprehensive understanding of their collective impact. In addition, the expression levels and biological functions of KDM5 proteins differ in the different subtypes of BC. This provides important clues for investigating the occurrence and intervention mechanisms of BC, but at the same time may pose obstacles for the targeting of the KDM5 family for treating BC.

In recent decade, the KDM5 family has gained widespread attention in the literature. However, only one KDM5 inhibitor, the anti-hepatitis B virus agent GS-5801, has entered clinical trials [158]. This further emphasizes the necessity of studying the roles of KDM5 in BC and optimizing KDM5 inhibitors. Among the compounds listed in Table 2, metal complexes are more likely to exhibit promiscuity. Metal complexes can often interact with a variety of biological targets due to their versatile coordination chemistry and ability to bind to different biomolecules. Isothiazolone and thiotriazole compounds may also display promiscuity to some extent, but metal complexes generally have a higher potential for interacting with multiple targets. Nevertheless, a number of KDM5 inhibitors described in this review have displayed promising anti-BC activity in preclinical models. A rhodium-based complex **1** developed by our group exhibits comparable antitumor activity to the clinical drugs cisplatin and doxorubicin, but with significantly lower toxicity in a TNBC mouse model [145]. Compound **7** and its analogue patented by Gilead Sciences could suppress the proliferation of both trastuzumab-sensitive and trastuzumab-resistant HER2+ BC cells, and reduce tumorigenesis and tumor growth in vivo [122]. Moreover, several KDM5 inhibitors have displayed synergy with approved chemotherapy agents to significantly improve the efficacy of chemotherapy. For example, some KDM5 inhibitors sensitize endocrine-resistant cells to fulvestrant, while others mitigate adverse effects (Table 3). However, there are several challenges in the clinical development of KDM5 inhibitors. Currently, most KDM5 inhibitors target the catalytic activity of KDM5 by chelating Fe(II) or competing with 2-OG, making it difficult to achieve high selectivity due to the conserved catalytic core of KDM5 proteins. Furthermore, the majority of these inhibitors irreversibly suppress enzyme activity, which could potentially increase the risk-to-benefit ratio if off-target effects occur. To overcome these challenges, designing reversible KDM5 inhibitors that target specific sites based on the crystal structure of KDM5 family proteins can be a viable approach. Additionally, developing inhibitors that target the PHD1 domain to achieve conformational regulation of KDM5 shows promise. Artificial intelligence can play a crucial role in integrating databases from multiple sources to effectively identify drugs that specifically inhibit KDM5 members using approved clinical drugs. This approach can be faster, safer, and more cost-effective compared to developing entirely new molecules. Moreover, techniques such as nuclear magnetic resonance, small-angle scattering, and co-crystallization can provide a structural basis for understanding KDM5 proteins and their interactions with small molecule drugs. Furthermore, apart from KDM5, some other KDMs may also be aberrantly expressed in BC, such as LSD1 [20] and KDM4s [21], which suggests that developing dual-targeted agents against KDM5 and other KDMs is also a potential strategy for BC therapy.

**Table 3:** Preclinical KDM5 inhibitors combined with approved drugs for treating breast cancer

KDM5 inhibitors	Approved drugs	Benefits	Refs.
**3**	Decitabine	Improving the sensitivity of immune checkpoint blockade and promoting apoptosis of breast cancer cells.	148
**5**	Trastuzumab	Enhancing the inhibitory effect of trastuzumab on the proliferation of BT-474 cells.	149
**7**	Trastuzumab Lapatinib	Enhancing the inhibitory effect of trastuzumab or lapatinib on the proliferation of BT-474 and SK-BR-3cells.	122
**9**	Doxorubicin	Enhancing the inhibitory effect of doxorubicin on the proliferation of TNBC cells.	154
**12**	Fulvestrant	Enhancing the inhibitory effect of fulvestrant on the proliferation of breast cancer cells.	101
**13**	Fulvestrant	Enhancing the inhibitory effect of fulvestrant on the cell viability of breast cancer cells.	101
**14**	Fulvestrant	Significantly decreasing MCF-7 xenografts tumor volume.	157

In summary, this review has described the functions of KDM5 proteins in BC. Most evidence supports that KDM5A/B is a carcinogenic factor in BC, while KDM5C may have tumor suppressive functions. However, conflicting findings exist, potentially influenced by whether the genes targeted by KDM5 are oncogenic or anti-cancer, as well as the dominant mechanisms involved. As BC remains an incurable disease, delving deeper into the mechanisms of action of the KDM5 family holds crucial significance in offering valuable insights and directions for the treatment of BC.

## Data Availability

No datasets were generated or analysed during the current study.
